# Synthetic cartography for mapping biodiversity in the Mediterranean region: Sicily as a case study

**DOI:** 10.3897/phytokeys.109.28297

**Published:** 2018-10-16

**Authors:** Gianniantonio Domina, Giuseppe Venturella, Maria Letizia Gargano

**Affiliations:** 1 Department of Agricultural, Food and Forest Sciences, University of Palermo, Viale delle Scienze, Bldg. 4. I-90128 Palermo, Italy University of Palermo Palermo Italy; 2 Department of Earth and Marine Sciences, University of Palermo, Viale delle Scienze, Bldg. 16. I-90128 Palermo, Italy University of Palermo Palermo Italy

**Keywords:** plants, distribution, biological collections, spatial analysis, data analysis, GIS software

## Abstract

This paper proposes a new hierarchical land classification system for the mapping of species distribution at national or regional scales. Our integrative framework incorporates two hierarchical levels inferred from historical, climatic, geomorphological and geological attributes. The feasibility of this proposal is based on the use of historical collections and literature data, as well as on its ability to combine old low-precision data with more recent records of higher resolution. The system is set up for vascular plants, but it can also be used for other taxonomic groups. Furthermore, it has the potential for application to the whole Mediterranean region because it is based on information that is generally available in all Mediterranean countries. This model is tested with the distribution of *loci classici* of the Italian endemic plants occurring in Sicily.

## Introduction

Biodiversity mapping is widely considered as the basis for effective territorial and conservation planning. In the last 20 years, thanks in part to the development of GIS tools, the accuracy, quality and speed of realisation of such mapping has increased significantly (Lahti and Lampinen 1999). The first well known example in Europe of an atlas covering an entire regional flora was carried out for the British Isles (Perring and Walters 1962). The two main approaches adopted for mapping biological distribution data are, according to Pedrotti (2013), maps of dots and maps of polygons. Polygons can be regular or irregular in size and either freely arranged or according to a fixed grid. Dots in a fixed grid system can be assimilated to form regular polygons. Freely distributed dot mapping allows the identification of the geographical position of a taxon on a cartographic support with the highest possible precision (e.g. Barina 2017), but the output is not suitable for further statistical elaboration with commensurable units of investigation. Dot maps can be used when highly detailed distribution data are available, but for large-scale analysis, their conversion in a matrix is advisable. Maps comprising irregular polygons use portions of territory of different sizes and shapes as survey units, such as political-administrative borders or the limits of protected areas. For example, a large part of biodiversity data collection and processing activities in France is based on the 96 departments into which its territory is subdivided (e.g. Prelli and Boudrie 1992).

Although this approach has the advantage of ease of data collection regarding the areas investigated, subsequent statistical processing of the results may be difficult due to the irregular size of the geographic units. On the other hand, whereas the use of regular grids facilitates statistical analysis, e.g. the identification of grid cells of higher biodiversity (Raimondo 2000, Finnie et al. 2007, Carta et al. 2018), a large quantity of highly detailed data is required in order to provide reliable results. Grids can be arranged according to the U.T.M. (Universal Transverse Mercator) projection that subdivides Europe into units of 100 km^2^, with this main grid then further subdivided down to the desired level of detail (Raimondo and Venturella 1991, Domina et al. 2018). The first floristic maps of the entire European territory, based on its subdivision into survey units of 50 km^2^, were proposed in the Atlas Florae Europeae (Jalas and Suominen 1972); this system was later adapted to the whole Mediterranean region (Uotila et al. 2003).

Although subdivision into kilometric squares is currently little used, it does allow reference to areas of equal size. The Floristic Cartography Project of Central Europe was based on the geographical division of the territory (Ehrendorfer and Hammann 1965); thus, although its Operational Geographical Units (OGU) vary in size according to latitude, data can be located rapidly.

As a result of the increasing use of GPSs and easy access to geo-referenced maps (e.g. Google Earth), an almost global transition in the survey of primary biodiversity data has taken place from OGU-based systems to point systems (e.g. Bartolucci et al. 2016, Bedini et al. 2016).

Nevertheless, herbaria and other historical collections remain one of the main sources of primary biodiversity data that should not be neglected. This is true both for historical data from countries that are also covered by updated surveys, for the study of trends, but also for those countries for which data sets more than 50 years old remain the most abundant. Therefore, a problem has arisen concerning the use of low resolution data either on their own or together with more detailed datasets.

Brundu et al. (2017), in the framework of the project for the identification of the *loci classici* of the flora endemic to Italy (Domina et al. 2012, Peruzzi et al. 2015), highlighted the problem of managing historical herbarium data, but ultimately limited their considerations only to data that could be projected on to a GIS system as reliable points. Within this context, this paper presents a hierarchical approach specifically designed for the mapping and comparison of plan distribution data at different scales, on behalf of environmental analysis. The results are a synthetic environmental cartography, with the purpose of delimiting of spatial sets, which are groupings of unitary areas of analysis characterised by homogeneous variables. The system proposed here integrates floristic data with existing information from various environmental disciplines, including geology, bioclimatology, vegetation science and soil science (Blasi 1995).

### A proposal for classifying and mapping floristic data

The key problem in organising a classification system based on biologically homogeneous areas is the development of the criteria used to identify homogeneity at different spatial scales (disregarding time). In fact, different natural processes occur on different temporal scales (Klijn and de Haes 1994) and thus the use of fixed study areas also allows such a comparison of these phenomena.

Typically, the land attributes used to classify homogeneous areas include flora, climate, lithology, geomorphology, human activities, soil, vegetation and fauna (Forman and Godron 1986, Zonneveld 1995). As the weight selected for each of these different attributes determines the delimitation of the homogeneous areas (Bunce et al. 1996), arbitrary elements may be introduced even in objective approaches. The system proposed here is thus based largely on an intuitive, divisive approach, using generally available data with superimposed maps.

## Materials and methods

### Study area

Sicily is located at the centre of the Mediterranean Basin and is considered one of its most important biodiversity hotspots (Medail and Quezel 1997). The total area of Sicily is about 25,700 km^2^, of which approximately 61.4% is hilly, 24.5% is mountainous and the remaining 14.1% consists of alluvial plains.

Sicily is surrounded by more than 300 smaller islands and islets, some of which are only rocks isolated from the mainland, on which plants permanently occur. According to Basilone (2018), eight main geological complexes can be distinguished in Sicily: Continental deposition clastic, Volcanic, Clayey-marly, Evaporitic, Sandstone-clayey-calcareous, Carbonate, Phyllitic and shale-crystalline. Bazan et al. (2015) recognise 11 different bioclimatic belts, ranging from Upper lnframediterranean to Upper Cryomediterranean. This variation in geology, morphology, climate and land use is largely responsible for Sicily’s extremely rich biological diversity. Indeed, its currently known vascular flora, 3224 specific and subspecific taxa, include more than 13% of the Italian endemic taxa (Bartolucci et al. 2018, Galasso et al. 2018).

### Mapping

A geographical information system was used to collate the information and to draw the maps (QGIS 3.0, https://www.qgis.org). The base maps are, in order: a 20 × 20 m Digital Terrain Model (http://wms.pcn.minambiente.it); a map of regional water bodies (http://www.pcn.minambiente.it/arcgis/services); a structural map of Sicily (Accaino et al. 2011); a lithological map of Sicily (Fierotti 1997); maps of temperature (Drago 2005), rainfall (Drago 2005), bioclimate (Bazan et al. 2015) and land use (Corine Land Cover, level 1, http://www.sitr.regione.sicilia.it/geoportale).

**Figure 1.**
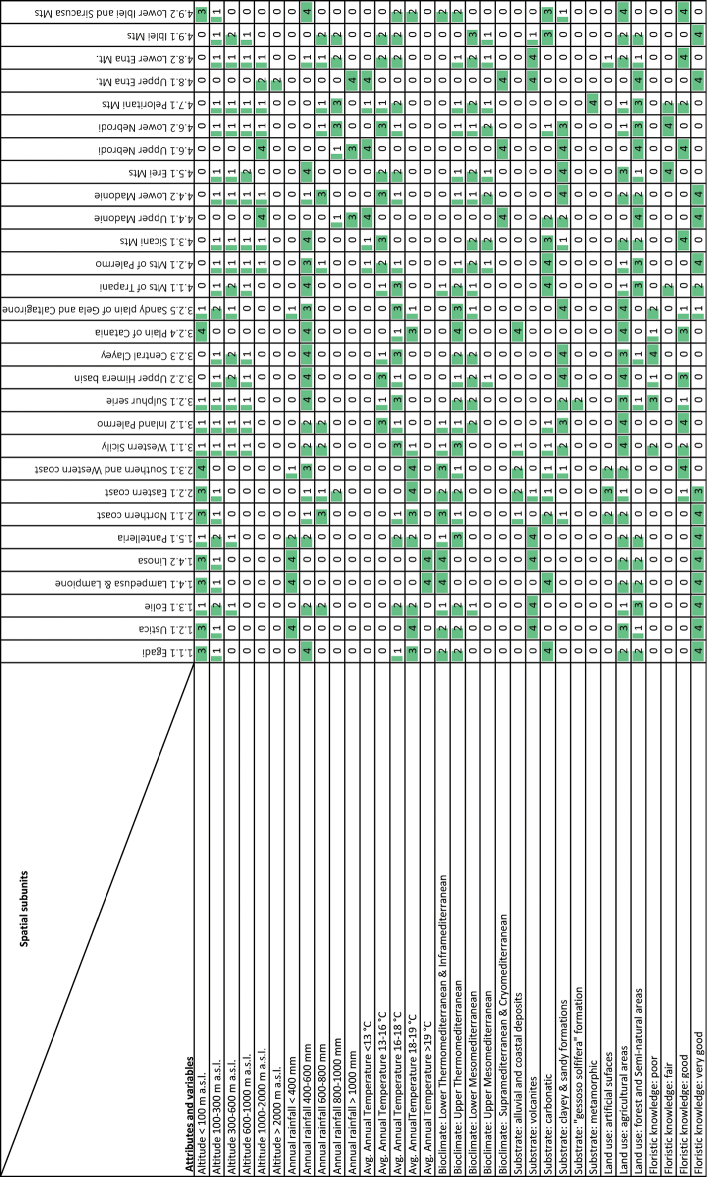
Matrix in numerical and graphical form with the data attributes and variables relative to the elementary spatial subunits.

Area boundaries were traced according to level curves, rivers and geological structures. We started tracing the outlines of the areas historically visited by botanists following Raimondo et al. (2005). Subsequently, these and the remaining areas were divided by tracing the boundaries according to altitude, bioclimatology and geology (Table 1). We used a matrix according to Martinelli (2018) for synthetic cartography, which is obtained from an array of spatial data with columns representing the elementary spatial units of analysis and rows their attributes. The ordinate matrix with the attributes and variables extracted from the thematic maps of analysis reports the class; this matrix is transcribed into a graphical form, as a grid, with cells, which are filled by proportional sizes established in five classes (Fig. 1). The classes range from 0 to 4 giving 0 as a null value, 1 for 25%, 2 for 50%, 3 for 75% and 4 for 100% of occupied surface. The identified areas are homogenous concerning the selected attributes.

**Table 1.** Subdivision of the Sicilian territory into groups, units and subunits.

Groups	Units	Subunits
1. Islands	1.1 Egadi	1.1.1 Egadi
	1.2 Ustica	1.2.1 Ustica
	1.3 Eolie	1.3.1 Eolie
	1.4 Pelagie	1.4.1 Lampedusa & Lampione
		1.4.2 Linosa
	1.5 Pantelleria	1.5.1 Pantelleria
2. Coasts	2.1 Northern coast	2.1.1 Northern coast
	2.2 Eastern coast	2.2.1 Eastern coast
	2.3 Southern and Western coast	2.3.1 Southern and Western coast
3. Hills and plains	3.1 Western Sicily and inland Palermo	3.1.1 Western Sicily
		3.1.2 Inland Palermo
	3.2 Hilly inland	3.2.1 Sulphur serie
		3.2.2 Upper Himera basin
		3.2.3 Central Clayey
		3.2.4 Plain of Catania
		3.2.5 Sandy plain of Gela and Caltagirone
4. Mountain Systems	4.1 Mts of Trapani	4.1.1 Mts of Trapani
	4.2 Mts of Palermo	4.2.1 Mts of Palermo
	4.3 Sicani Mts	4.3.1 Sicani Mts
	4.4 Madonie Mts	4.4.1 Upper Madonie
		4.4.2 Lower Madonie
	4.5 Erei Mts	4.5.1 Erei Mts
	4.6 Nebrodi Mts	4.6.1 Upper Nebrodi
		4.6.2 Lower Nebrodi
	4.7 Peloritani Mts	4.7.1 Peloritani Mts
	4.8 Etna Mt.	4.8.1 Upper Etna Mt.
		4.8.2 Lower Etna Mt.
	4.9 Iblei and Siracusa Mts	4.9.1 Iblei Mts
		4.9.2 Lower Iblei and Siracusa Mts

In order to verify the adequacy and the advantage of the proposed model, we used the dataset of the *loci classici* (localities reported in protologues) of the Italian endemics described from Sicily (Domina et al. 2012, Peruzzi et al. 2015, Brundu et al. 2017) and compared our results with those obtained in Brundu et al. (2017). The endemics, in fact, have been considered the most sensible element of the flora of a region and provide valid indications of the whole (Brundu et al. 2017). We projected the data collected for the project “Italian Loci Classici” on the obtained map. The loci with high geographic detail were projected using their exact coordinates. The loci with low geographic detail were projected using the coordinates of their toponyms. For this project, the geographic locations reported in the protologues or in the types of plants endemic to Italy were mapped reporting their geographical accuracy. In Brundu et al. (2017), all the type localities with low geographic accuracy (>10 km) were discarded. Here we used the complete Sicilian dataset excluding only data generically referred to the whole Sicily.

## Results

### Area subdivision

**Figure 2.**
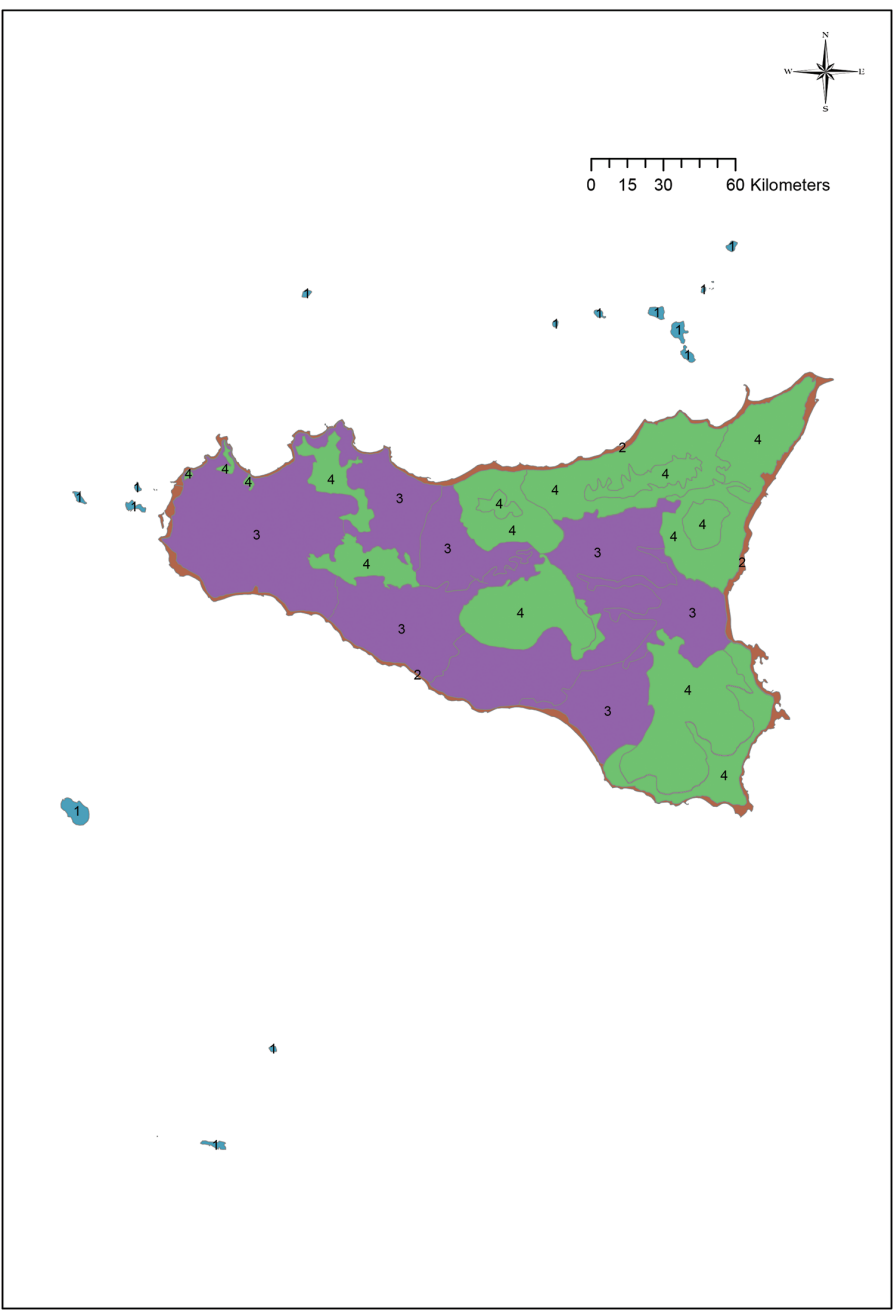
The four groups into which Sicily is divided, as reported in Table 1.

**Figure 3.**
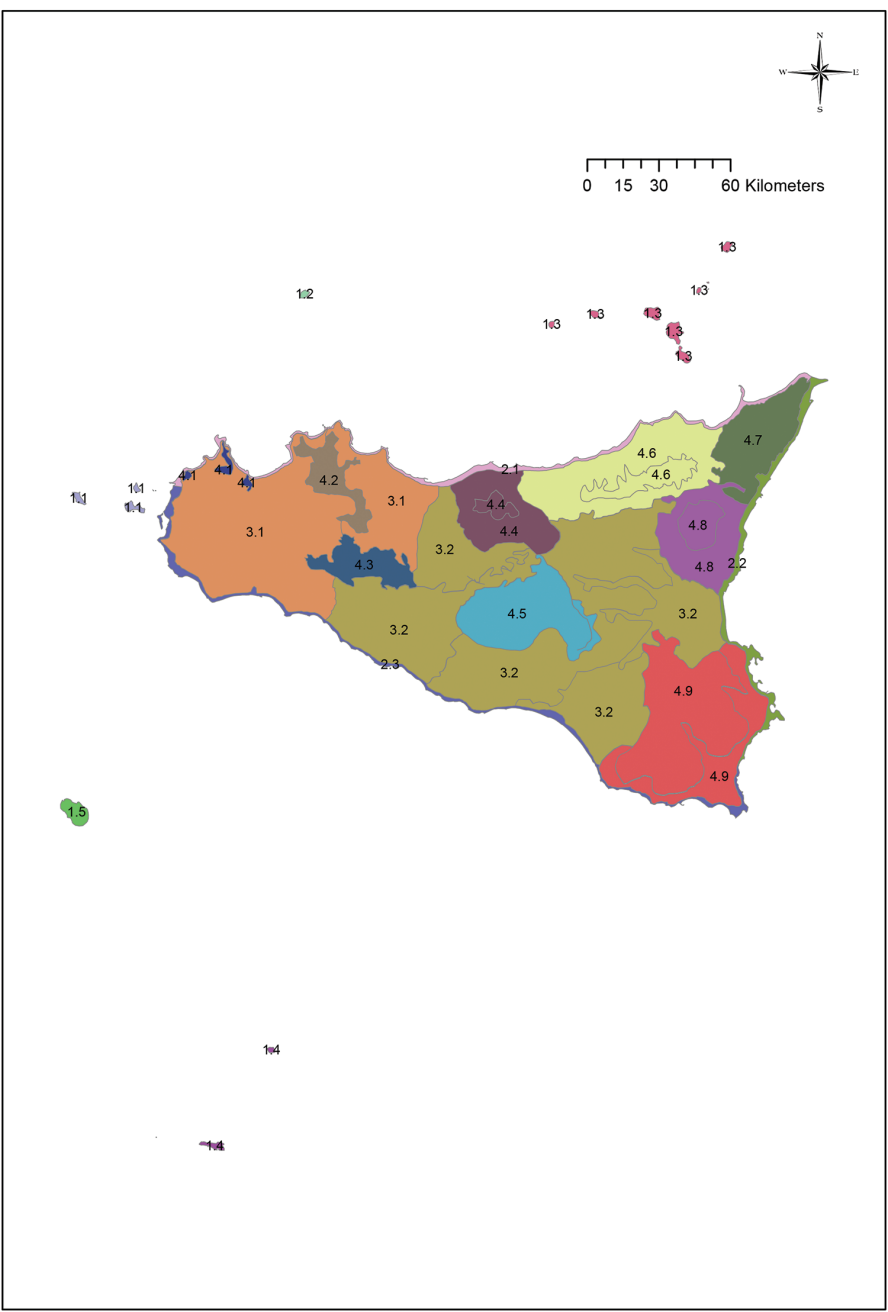
The 19 units into which Sicily is divided, as reported in Table 1.

**Figure 4.**
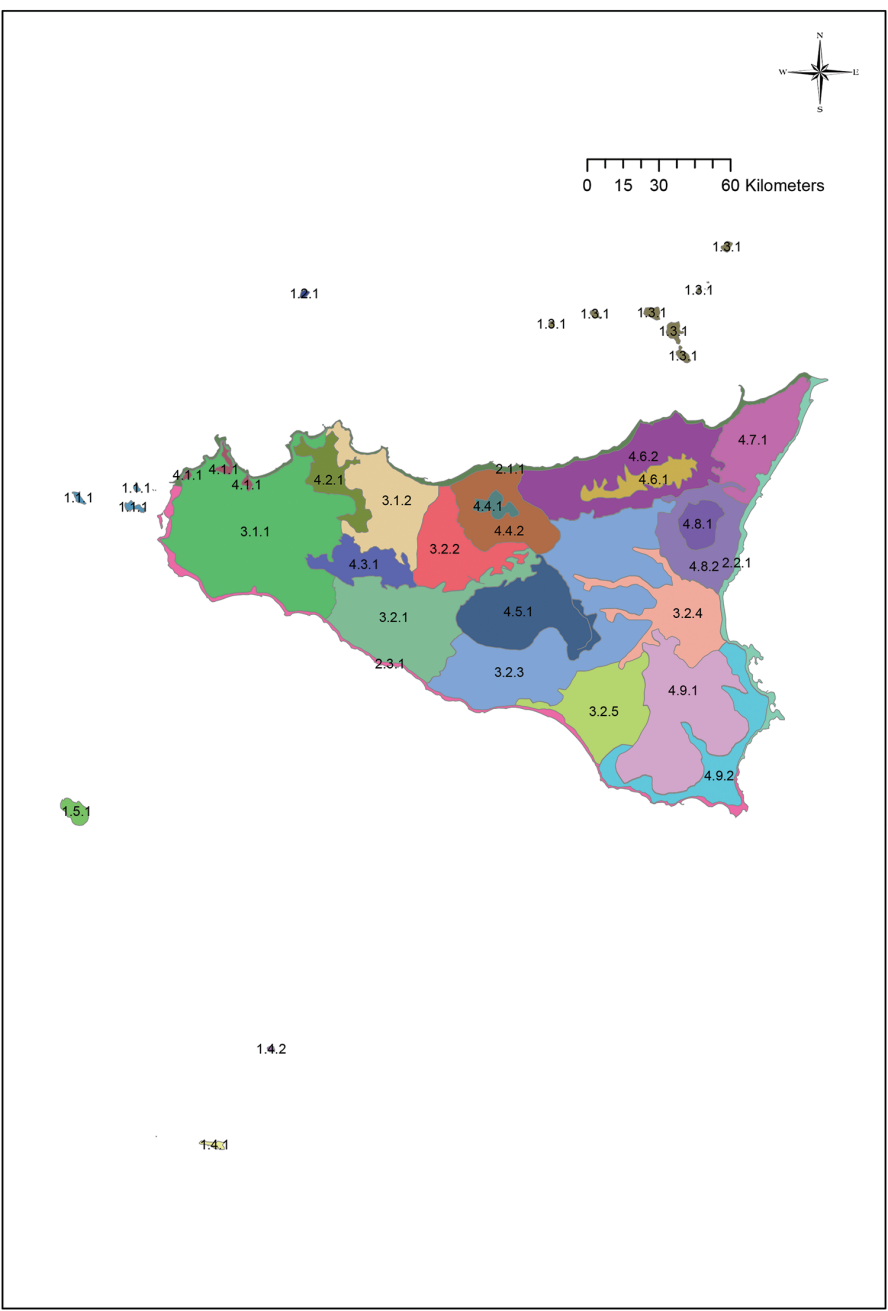
The 29 sub-units into which Sicily is divided, as reported in Table 1.

Overall, the Sicilian territory can be divided into four main groups: islands, coasts, hills and plains and mountains (Fig. 2). These four groups can be subdivided into 19 units (Fig. 3) that can in turn be further split into 29 subunits (Fig. 4) (Table 1). A detailed description and presentation of these subunits is beyond the scope of the present paper. Unit delimitation is based on major morphological features, while that of subunits is based mainly on altitude or geological substrata that determine different floristic contingents. The main islands surrounding Sicily include two isolated volcanic islands (Ustica and Pantelleria) and three archipelagoes. Egadi and Eolie archipelagoes consist of islands that are homogeneous in terms of substrate (carbonatic and volcanic, respectively) and thus, despite some of the islands hosting narrow endemic species, these archipelagoes are not subdivided further. In contrast, the Pelagie Archipelago comprises two carbonatic islands and one volcanic island whose variation in substrate has resulted in large differences in their floras; for this reason these islands are divided into two subunits (Lampedusa & Lampione and Linosa).

Coasts are subdivided according to the nature of their substratum and morphology (mainly sandy in the south and rocky in the north). This diversity is reflected in the presence of species exclusive to coastal cliffs (class *Chitmo-Limonietea* Br.-Bl.) or sandy dunes (class *Cakileteamaritimae* Br.-Bl. & Tüxen). The Sicilian hinterland is more homogeneous from a floristic point of view and thus can be considered as a single unit. In contrast, the subdivision of the hilly inland on the basis of substrate allows, for example, to highlight areas containing species restricted to the local gypsum-sulphur formation. Although the plant landscape of the region is largely anthropogenic (Gianguzzi et al. 2016), variation in soil types can also be used to distinguish areas traditionally devoted to agriculture, such as the Plain of Catania, where non-native communities are dominant. The main mountain chains are identified as the following distinct mapping units: Trapani, Palermo, Sicani, Madonie, Nebrodi, Etna, Peloritani and Iblei. The highest mountain ranges are divided into upper and lower subunits on the basis of the lower altitudinal limit of the beech forest (e.g. 1200 m a.s.l. in the Madonie Mts). The upper part of these particular mountains is characterised by unique bioclimatic features and hosts the highest percentage of Italian endemic species on the island (Baiamonte et al. 2015).

### Model test

**Figure 5.**
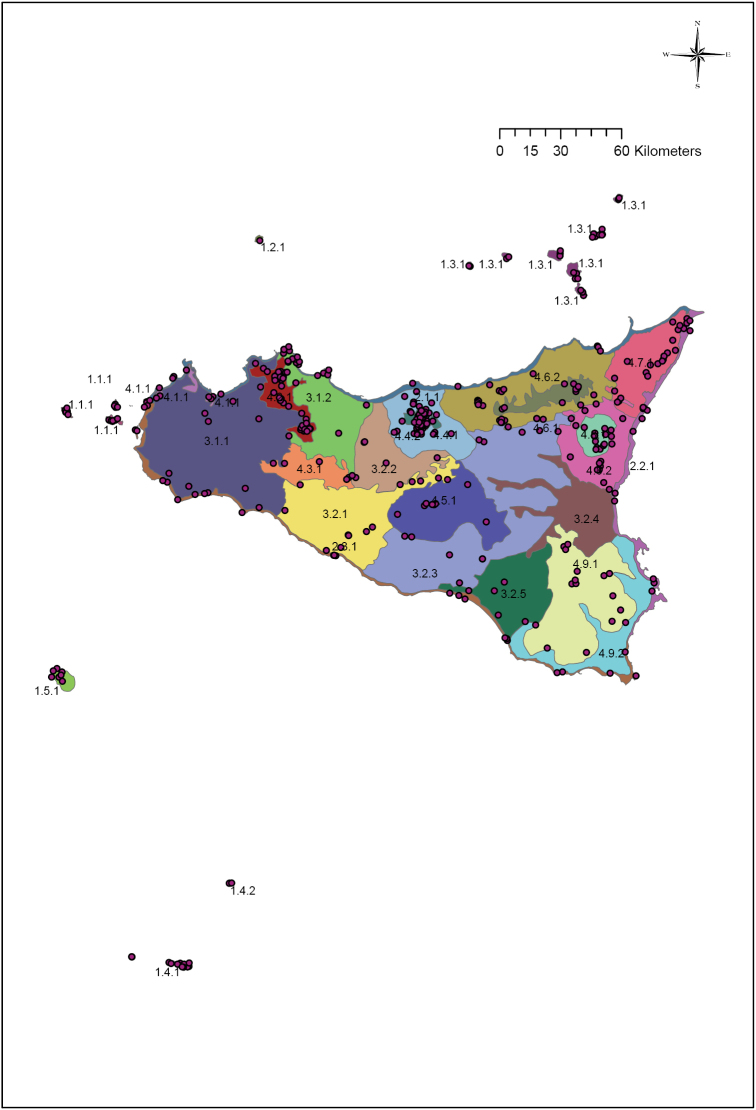
Projection on the sub-units of the loci classici of the Italian endemics described from Siciliy (Brundu et al. 2017).

In total, we projected 472 points on the map (Fig. 5), representing a gain of 111 points (31%) over the work of Brundu et al. (2017) where only 361 points were included. All the points have been attributed to single areas without ambiguity. This allows us to distinguish areas richer in *loci classici* (i.e., likely richer in endemics) and, therefore, to make biogeographical speculations.

Overall (Table 2), the mountain areas host more loci than the other areas identified in the region (277 vs. 195). Islands, coasts and hills have a comparable number of loci (71, 59 and 65, respectively). The richest area overall is the upper part of the Madonie mountains (74 loci) followed by the mountains around Palermo (53). The Nebrodi Mountains, siliceous and rounded, are richer on the lower belt (23) than on the summit (9). The Etna Mountain is about equally rich on the lower (16) and the upper belt (18). As pointed out by Mazzola et al. (2001), on the basis of the analysis of the biological and chorological spectra of the whole vascular floras, the Aeolian archipelago is the richest archipelago (28 loci) while the poorest island is Ustica (only 1 locus).

**Table 2.** Occurrence of *loci classici* of the Italian endemic flora in the recognised groups, units and subunits.

Groups	Loci	Units	Loci	Subunits	Loci
1. Islands	71	1.1 Egadi	17	1.1.1 Egadi	17
		1.2 Ustica	1	1.2.1 Ustica	1
		1.3 Eolie	28	1.3.1 Eolie	28
		1.4 Pelagie	18	1.4.1 Lampedusa & Lampione	16
				1.4.2 Linosa	2
		1.5 Pantelleria	7	1.5.1 Pantelleria	7
2. Coasts	59	2.1 Northern	21	2.1.1 Northern	21
		2.2 Eastern	14	2.2.1 Eastern	14
		2.3 Southern and Western	24	2.3.1 Southern and Western	24
3. Hills and plains	65	3.1 West Sicily and Palermo’s inland	31	3.1.1 West Sicily	15
				3.1.2 Palermo’s inland	16
		3.2 Hilly inland	34	3.2.1 Sulphur serie	12
				3.2.2 Upper Himera basin	6
				3.2.3 Central Clayey	11
				3.2.4 Plain of Catania	0
				3.2.5 Sandy plain of Gela and Caltagirone	5
4. Mountain Systems	277	4.1 Mts of Trapani	4	4.1.1 Mts of Trapani	4
		4.2 Mts of Palermo	53	4.2.1 Mts of Palermo	53
		4.3 Sicani Mts	6	4.3.1 Sicani Mts	6
		4.4 Madonie Mts	101	4.4.1 Upper Madonie	74
				4.4.2 Lower Madonie	27
		4.5 Erei Mts	9	4.5.1 Erei Mts	9
		4.6 Nebrodi Mts	32	4.6.1 Upper Nebrodi	9
				4.6.2 Lower Nebrodi	23
		4.7 Peloritani Mts	20	4.7.1 Peloritani Mts	20
		4.8 Etna Mt.	34	4.8.1 Upper Etna Mt.	18
				4.8.2 Lower Etna Mt.	16
		4.9 Iblei and Siracusa Mts	18	4.9.1 Iblei Mts	14
				4.9.2 Lower Iblei and Siracusa Mts	4

## Discussion and Conclusion

Practical methods for tracing homogeneous areas are required in all types of studies at various scales. The most topical issue facing regional landscape planning is the identification of homogeneous areas to which individualised management should be applied (Blasi et al. 2000).

Brullo et al. (1995) proposed a phytogeographic subdivision of Sicily into 2 sectors, 4 sub-sectors and 15 districts based on the presence of groups of endemic plants. This proposal is partially congruent with the 17 units identified here. However, such floristic districts, although functional from a large-scale biogeographical point of view, are less useful on the regional scale due to variations in altitude and geology.

The land classification, here proposed, integrates biotic and abiotic parameters at different scales. Its units and subunits are easily recognisable and comprehensible and can be used effectively at supra-national and regional scales for biodiversity, historical and ecological analysis. This approach also enables easy data presentation and allows the comparison of time series of historical and modern data. In addition, it is a method that facilitates rapid analysis on a regional scale in areas for which no detailed data are available, as is still the case for large portions of North Africa.

Our suggestion is to project all the available data in the GIS system, indicating their accuracy, date and reliability, using the geographical coordinates of the selected points or the toponyms to which they refer. More detailed data can be easily lowered to the accuracy level proposed here. Subsequent extrapolation for spatial or statistical analyses can then be performed on different sub-sets of data or in different layers depending on the required output. Point data collection and analysis in terms of administrative areas are used in the Wikiplantbase project for the mapping of plant species in some regions of Italy (Peruzzi et al. 2017). Our proposal allows the projection of historical data at a low level of detail, making them available for various analyses. Although these data are often overlooked, due to a lack of proper spatialisation, they may in fact be useful for regional floras and national checklists.
